# Association between serum free fatty acid levels and nonalcoholic fatty liver disease: a cross-sectional study

**DOI:** 10.1038/srep05832

**Published:** 2014-07-25

**Authors:** Juanwen Zhang, Ying Zhao, Chengfu Xu, Yani Hong, Huanle Lu, Jianping Wu, Yu Chen

**Affiliations:** 1 Department of Laboratory Medicine, The First Affiliated Hospital, College of Medicine, Zhejiang University, 79 Qingchun Road, Hangzhou 310003, China; 2 Department of Gastroenterology, The First Affiliated Hospital, College of Medicine, Zhejiang University, 79 Qingchun Road, Hangzhou 310003, China; 3 These authors contributed equally to this work.

## Abstract

High serum free fatty acid (FFA) levels are associated with metabolic syndrome (MS). This study aimed to assess the association of fasting serum FFAs with nonalcoholic fatty liver disease (NAFLD) in a Chinese population. A total of 840 subjects fulfilled the diagnostic criteria of NAFLD and 331 healthy control participants were enrolled in this cross-sectional study. Fasting serum FFA levels and other clinical and laboratory parameters were measured. NAFLD patients had significantly higher serum FFA levels than controls (*P* < 0.001). Serum FFA levels were significantly and positively correlated with parameters of MS, inflammation indexes, and markers of hepatocellular damage. Elevated serum FFA levels were found in NAFLD subjects with individual components of MS (obesity, hypertriglyceridaemia, and hyperglycaemia). Stepwise regression showed that serum FFA levels were an independent factor predicting advanced fibrosis (FIB-4 ≥ 1.3) in NAFLD patients. Serum FFA levels correlated with NAFLD and could be used as an indicator for predicting advanced fibrosis in NAFLD patients.

Nonalcoholic fatty liver disease (NAFLD) refers to a wide spectrum of chronic liver disorders, from simple steatosis to nonalcoholic steatohepatitis (NASH) and advanced fibrosis and cirrhosis without significant ethanol consumption^1^,^2^. Indeed, simple steatosis is generally stable and does not evolve into NASH in most cases. Only a minority of individuals, those with NASH, are prone to the risk of fibrosis and cirrhosis^3^. NAFLD has recently been recognised as a major public health problem, affecting as much as 20% of the general population in China over the past few decades^4^,^5^. The etiology of NAFLD reflects complex interactions between genetic, neurohumoral, metabolic and stress-related factors^6^,^7^. The liver plays a principal role in lipid metabolic pathways by taking up serum free fatty acid (FFA), and manufacturing, storing, and transporting lipid metabolites^8^. The accumulation of lipids, mainly triacylglycerol (TAG), in hepatocytes is the hallmark feature of the pathogenesis of NAFLD^9^. Donnelly et al. reported that the circulating nonesterified fatty acid pool contributed to the majority of the FFA that flow to the liver and constituted the bulk of the fasting liver TAG pool^10^.

Metabolic syndrome (MS) is the term given to a cluster of risk factors for cardiovascular disease, including abdominal obesity, diabetes mellitus with raised fasting plasma glucose, raised blood pressure and dyslipidaemia^11^. Over the past two decades, a striking increase in the prevalence of MS worldwide has taken place along with the global epidemic of obesity^12^. This constellation of metabolic abnormalities is also becoming increasingly common in China, as shown by emerging prevalence data^13^. MS has been associated with an increased risk of NAFLD and cardiovascular disease morbidity and mortality, resulting in an increased economic burden on society^14^. The most widely accepted mechanism underlying MS is insulin resistance (IR)^15^. Over the past few years, an association between increased fatty acid flux and MS has been well demonstrated^16^,^17^. IR results in excessive flux of fatty acid as a result of unopposed adipose tissue lipolysis^18^. Accumulation of FFA can further increase IR by modulating insulin receptor expression and post-receptor signalling^19^.

NAFLD is considered to be the hepatic manifestation of metabolic syndrome, sharing a causative factor in IR^20^. The association between NAFLD and FFA level is controversial in the literature. Some studies have focused on the same lipotoxic properties of FFA, however, a recent in vitro study proposed that the cellular and metabolic effects of FFA on hepatocytes vary depending on their composition^21^,^22^,^23^. However, there are limited studies investigating serum FFA levels in patients with NAFLD. Serum alanine aminotransferase (ALT), aspartate aminotransferase (AST), and glutamyltransferase (GGT) are closely related to NAFLD and may act as markers for the severity of liver damage^24^,^25^. MS and inflammation are well-established risks factor for NAFLD^26^,^27^. We hypothesise that analysis of the relationship between serum FFA levels and parameters of metabolic syndrome (body mass index, BMI; systolic blood pressure, SBP; triglyceride, TG; total cholesterol, TC; fasting plasma glucose, FPG), inflammatory indexes (sialic acid, SA; high-sensitivity C-reactive protein, hsCRP; white blood cells, WBC) and markers of hepatocellular damage (ALT, AST and GGT) may indirectly lead to a further understanding of serum FFA levels and NAFLD. This cross-sectional study aimed to characterise the relationship between changes in serum FFA and NAFLD in a Chinese population.

## Methods

### Subjects

The study initially enrolled 920 patients diagnosed with fatty liver based on abdominal ultrasonography. Subjects who met the following criteria were excluded: (i) those with alcohol consumption > 140 g/week for men and > 70 g/week for women (n = 20); (ii) those with a history of viral hepatitis (n = 46), autoimmune hepatitis or other forms of chronic liver disease (n = 14). The remaining 840 patients with NAFLD (mean age: 46.1 ± 12.2 years; female: 239; male: 601) and 331 age- and gender-matched healthy subjects (mean age: 47.0 ± 10.7 years; female: 96; male: 235) were used in the current analyses. Informed consent was obtained from all the subjects and the study was approved by the ethics committee of the first affiliated hospital of the medical college at Zhejiang university in China.

### Clinical and anthropometric parameters

The clinical examinations were conducted in the morning after an overnight fast. Weight, height and blood pressure (SBP; DBP, diastolic blood pressure) were measured, and BMI was calculated as weight in kilograms divided by height in meters squared. FIB-4 index was calculated according to the following equation: 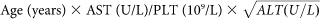
28. Liver ultrasound examinations were performed by experienced radiologists who were unaware of the clinical and laboratory data, using a Toshiba Nemio 20 sonography machine with a 3.5-MHz probe (Toshiba, Tokyo, Japan).

### Biochemical analyses

Fasting whole blood samples were obtained from an antecubital vein and blood samples were used for the analysis of the haematological index and biochemical values. All samples were analysed by specialised clinical laboratory medical personnel. The laboratory parameters included measurement of ALT, AST, GGT, creatine kinase (CK), TG, TC, HDL-C, low-density lipoprotein cholesterol (LDL-C), uric acid (UA), FPG, haemoglobin A_1c_ (HbA1c), homocysteine (Hcy), SA, hsCRP, WBC, pancreatic lipase (P-LIP), amylase (AMY), FFA, haemoglobin (HGB), platelets (PLT) and red blood cell distribution width (RDW). All biochemical values were measured using a Hitachi 7600 clinical analyser (Hitachi, Tokyo, Japan) and Sysmex XE-2100 auto-analyser (Sysmex, Kobe, Japan) using standard methods.

### Diagnostic criteria for NAFLD and MS

Hepatic steatosis was diagnosed according to the guidelines established for the diagnosis and treatment of NAFLD issued by the Fatty Liver Disease Study Group of the Chinese Liver Disease Association^29^. Specifically, hepatic steatosis was diagnosed according to characteristic echo patterns, such as diffuse hyperechogenicity of the liver relative to the kidneys, ultrasound beam attenuation, and poor visualisation of intrahepatic structures. The prevalence of MS differs widely in varying studies according to the definition criteria used and the population sample studied^30^,^31^. To adopt an ethnicity-specific value of waist circumference, the International Diabetes Federation (IDF) has proposed regional definitions of central obesity according to the characteristics of Chinese people. According to IDF guidelines, for a person to be defined as having MS they must have three or more of the following: central obesity (defined as a waist circumference > 90 cm for Chinese men and > 80 cm for Chinese women); BMI > 25 kg/m^2^; raised circulating triglyceride levels (defined as triglycerides ≥ 1.7 mmol/L) or specific treatment for this lipid abnormality; reduced HDL-C levels (defined as HDL-C < 1.03 mmol/L in male patients and < 1.29 mmol/L in female patients); raised blood pressure (defined as SBP ≥ 130 mm Hg or DBP ≥ 85 mm Hg) or treatment for previously diagnosed hypertension; and raised FPG (defined as FPG ≥ 5.6 mmol/L) or previously diagnosed type 2 diabetes.

### Data and statistical analysis

Statistical analyses were performed using SPSS, version 16 (SPSS, Chicago, IL, USA). Data are presented as the mean ± standard deviation when data were found to be normally distributed or as the median if the distribution was skewed. Differences between groups were analysed using the Student's *t*-test or the Mann–Whitney *U* test. Spearman correlation analysis was used to examine the correlation between serum FFA levels and clinical and laboratory parameters. The parameters for multivariate logistic regression were selected by univariate regression analysis.

### Ethics Statement

This study was approved by the Hospital Ethics Committee and was performed in accordance with the Helsinki Declaration.

## Results

### Characteristics of Subjects

Demographic and biochemical characteristics of study participants are summarised in Table 1. We found that patients with NAFLD had significant differences in terms of BMI, SBP, DBP, ALT, AST, GGT, CK, TG, TC, HDL-C, LDL-C, FPG, UA, HbA_1C_, Hcy, Fe, SA, hsCRP, WBC, P-LIP, AMY, HGB, PLT and RDW compared with controls. Specifically, the levels of these parameters were less favourable in subjects with NAFLD compared with the controls. Consistent with our hypothesis, we found that serum FFA levels were markedly higher in patients with NAFLD than in the controls (median 0.65 mmol/L *vs.* 0.45 mmol/L; *P* < 0.001). Characteristics of the NAFLD patients according to their diabetic status are presented in Table 2. The diabetic NAFLD group had higher GGT, TG, FPG, UA, HbA_1C_, SA and WBC levels and a lower AMY level. Notably, significantly higher FFA levels were observed in the NAFLD subjects with diabetes.

### Association between serum FFA levels and NAFLD

Our results show that serum FFA levels were significantly and positively correlated with parameters of MS [body mass index (r = 0.189, *P* < 0.001), triglyceride (r = 0.338, *P* < 0.001), total cholesterol (r = 0.169, *P* < 0.001) and fasting plasma glucose (r = 0.205, *P* < 0.001)], inflammatory indexes [sialic acid (r = 0.196, *P* < 0.001), high-sensitivity C-reactive protein (r = 0.145, *P* = 0.001) and white blood cell (r = 0.177, *P* < 0.001)] and markers of hepatocellular damage [(alanine aminotransferase (r = 0.157, *P* < 0.001), aspertate aminotransferase (r = 0.182, *P* < 0.001) and glutamyltransferase (r = 0.179, *P* < 0.001)], and negatively correlated with high-density lipoprotein cholesterol levels [(r = −0.172, *P* < 0.001)]. MS and inflammation are two major factors that are closely associated with NAFLD^23^. The present observation suggest that they may act as cofactors for the link between serum FFA levels and NAFLD.

**Figure 1:**
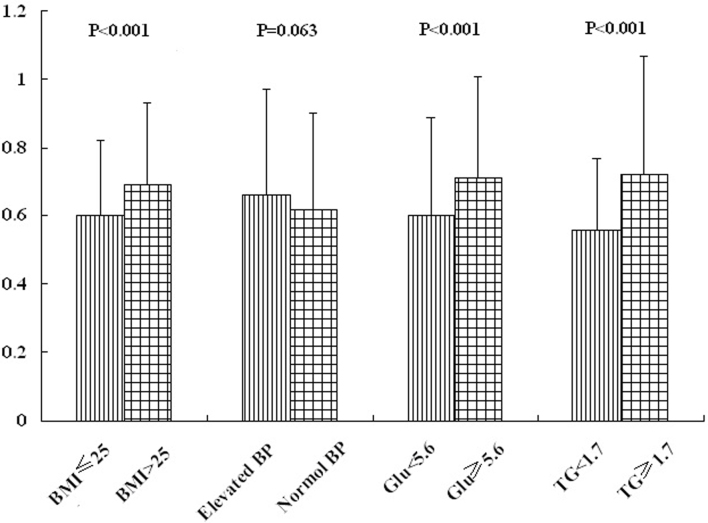
Serum FFA levels according to characteristics of metabolic syndrome.

As shown in Figure 1, serum FFA levels were significantly higher in obese subjects (BMI > 25 kg/m^2^) than in lean subjects (BMI ≤ 25 kg/m^2^). However, there was no significant difference when subjects were classified according to blood pressure. The group with higher FPG levels (FPG ≥ 5.6 mmol/L) showed increased serum FFA levels. Similarly, the FFA levels were increased in the higher TG group (TG ≥ 1.7 mmol/L) compared with the lower TG group (TG < 1.7 mmol/L).

Serum FFA levels in the group with FIB-4 ≥ 1.3 were significantly higher than those in the group with FIB-4 < 1.3 (median 0.72 mmol/L *vs*. 0.58 mmol/L; *P* < 0.001). We used univariate regression to analyze the odds and p-values (shown in Table 3) with forward selection, and we subsequently considered FFA, age, gender, BMI, γ-GT, ALT, hsCRP, and presence of diabetes (all p < 0.05) within the multivariate regression analysis. The results of unadjusted and adjusted multivariate logistic regression analysis models are shown in Table 4. Following adjustment for age, gender, BMI, γ-GT, ALT, hsCRP, and presence of diabetes (all *P* < 0.05) selected by stepwise regression, showed that serum FFA levels were an independent factor predicting advanced fibrosis (FIB-4 ≥ 1.3) in NAFLD participants.

## Discussion

This is one of the few studies performed to date addressing changes in blood biochemical parameters in a large cohort (n = 840) of subjects of the same ethnicity (Chinese) with NAFLD. The results suggest that serum FFA levels are strongly association with NAFLD. First, NAFLD patients had increased serum FFA levels, and these levels were positively correlated with parameters of MS (BMI, TG, TC and FPG), indexes of inflammation (SA, hsCRP and WBC) and markers of hepatocellular damage (ALT, AST and GGT). Second, NAFLD subjects with diabetes had higher serum FFA levels. Third, to obtain a better understanding of the association between serum FFA levels and NAFLD, all subjects with NAFLD were classified according to components of MS. This subgroup analysis indicated elevated serum FFA levels were significantly associated with individual components of MS (obesity, hypertriglyceridaemia and hyperglycaemia). Furthermore, logistic regression analysis showed that serum FFA levels were an independent factor predicting advanced fibrosis (FIB-4 ≥ 1.3) in NAFLD subjects.

One of the possible explanations for the increased FFA levels in NAFLD patients is insulin resistance^32^,^33^. Insulin resistance is well known as a pathophysiological hallmark of NAFLD^34^. Fasting serum FFA primarily originate from lipolysis in adipose tissue. The human body mobilises stored fat by catalysis of hormone sensitive lipase, which hydrolyses the fat into glycerol and fatty acids in the fasted state^35^. Insulin induces the dephosphorylation of hormone-sensitive lipase by reducing the concentration of cyclic adenosine monophosphate (cAMP) and activating phosphoprotein phosphatase, thus inhibiting lipase activity and decreasing fat hydrolysis^36^. It has been extensively documented that insulin resistance results in an increased flux of FFA.

The other possible mechanism linking serum FFA with NAFLD is the impaired ability of the liver to export or utilise FFA. Besides beta oxidation, the majority of FFA entering the hepatocyte are used for the synthesis of triglyceride, which then becomes a constituent of very low density lipoprotein (VLDL) particles that are exported into the blood^37^. It has been demonstrated that beta oxidation of FFA and export of VLDLs in the liver are impaired in NAFLD patients^38^. The elevated serum levels of FFA are therefore dependent on the imbalance between its origin and use in NAFLD.

The link between serum FFA levels and NAFLD suggested that FFA could play a pivotal role in the development of NAFLD^39^. Increased systemic oxidative stress in patients with NAFLD has long been recognised both in animal experiments and clinical studies^40^,^41^. FFA are responsible for development of hepatocellular apoptosis and injury, acting as a strong oxidant in several pathological states such as MS or type 2 diabetes^42^,^43^. High FFA levels also attenuate the insulin-mediated suppression of endogenous glucose production and gluconeogenesis, leading to the impairment of whole-body glucose tolerance^44^. High serum ALT, AST and GGT levels are associated with the more severe form of NAFLD, although low serum ALT, AST or GGT levels do not rule out the possibility of an advanced stage of NAFLD^45^,^46^. The correlation between FFA and markers of hepatocellular damage in the present study indirectly confirm the hypothesis that FFA significantly correlate with hepatocellular injury. Further studies are needed to clarify the detailed mechanism by which FFAs influence the progression of NAFLD.

NAFLD is a well-known risk factor for cardiovascular disease^47^. The association between FFA and NAFLD may provide a possible explanation for why NAFLD is closely related with cardiovascular disease. FFA can stimulate vascular smooth muscle proliferation and induce endothelial dysfunction, which may contribute to the cardiovascular events of NAFLD^48^. Furthermore, FFA have been implicated in the physiological mechanism of cardiovascular disease resulting from low-grade systemic inflammatory processes^49^,^50^. On the basis of our study, we can only propose a role of FFA in the aetiology of NAFLD and inflammation from a snapshot of the circulating FFA state. However, the question of whether or not elevated serum FFA are an unrelated phenomenon, a cause, or a consequence of NAFLD could not be determined from this cross-sectional study. Traditionally, NAFLD has been viewed as a spectrum that progresses from pure fatty liver through NASH to severe fibrosis, cirrhosis and eventually hepatocellular carcinoma^51^. According to recent views, uncomplicated steatosis and NASH tend to be considered as potentially unrelated disorders, not only based on histological definitions but also from pathophysiological evidence^52^,^53^. The identification of differences in serum FFA between simple steatosis and NASH in further studies may be important to clarify their precise interrelationship.

This study has some limitations. First, the diagnosis of NAFLD was based on ultrasonographic examination. Ultrasonography is not sensitive enough to detect mild steatosis, but overall it significantly correlates with both liver histology and metabolic derangements^54^. Given the acceptable sensitivity and specificity for detecting hepatic steatosis, ultrasonography is widely used in epidemiological studies of NAFLD^55^. Second, postprandial serum levels of FFA have not been examined in this study. Nevertheless, fasting serum FFA constitute the majority of fatty acids delivered to the liver and contribute to liver fat metabolism and accumulation in NAFLD. Third, we used the FIB-4 index for evaluating advanced fibrosis of NAFLD according the studies of Xun YH et al.^56^. FIB-4, a simple non-invasive index composed of readily available routine laboratory tests, can accurately predict advanced fibrosis and cirrhosis in patients with NAFLD. However, it is not sensitive enough to identify patients with a mild degree of fibrosis who are at risk of progression^57^,^58^.

In conclusion, our results demonstrate a significant correlation between serum FFA levels and NAFLD. Further research on the involvement of FFA in NAFLD will not only enhance our understanding of the development of NAFLD, but also benefit in the eventual development of new prevention and treatment strategies for the disease.

## Figures and Tables

**Table 1:** Demographic and biochemical characteristics of the study participants

Variable	Controls (n = 331)	NAFLD patients (n = 840)	*P* value
Age (yr)	47.0 ± 10.7	46.1 ± 12.2	0.29
Body mass index (kg/m^2^)	22.7 ± 2.6	26.3 ± 2.7	<0.001
Diabetes, n (%)	0	114(13.6%)	
Systolic blood pressure (mmHg)	124.1 ± 15.2	131.8 ± 15.4	<0.001
Diastolic blood pressure (mmHg)	75.4 ± 10.2	81.7 ± 15.2	0.001
Alanine aminotransferase (U/L)	16 (6–49)	29 (6–184)	<0.001
Aspertate aminotransferase (U/L)	19 (11–40)	24 (12–122)	<0.001
Glutamyltransferase (U/L)	18 (7–46)	34 (7.0–340.0)	<0.001
Creatine Kinase (U/L)	80(34–202)	91(17–330)	<0.001
Triglyceride (mmol/L)	0.97 (0.39–1.70)	1.66(0.34–20.30)	<0.001
Total cholesterol (mmol/L)	4.06 ± 0.58	5.06 ± 1.01	<0.001
High-density lipoprotein cholesterol (mmol/L)	1.39 ± 0.28	1.04 ± 0.26	<0.001
Low-density lipoprotein cholesterol (mmol/L)	2.41 ± 0.44	2.99 ± 0.56	<0.001
Fasting plasma glucose (mmol/L)	4.72 (3.81–6.01)	5.28(3.39–16.01)	<0.001
Uric acid (umol/L)	292.7 ± 60.1	364.3 ± 60.4	<0.001
Hemoglobin A1c (%)	5.4 (3.2–6.0)	5.7 (4.6–12.5)	<0.001
Homocysteine (umol/L)	8.0(3.1–13.1)	17.1(4.2–150.3)	<0.001
Fe (umol/L)	18.6(3.4–30.6)	23.3(8.1–106.1)	<0.001
Sialic acid (mg/dL)	54.1 ± 5.0	67.8 ± 6.5	<0.001
High-sensitivity C-reactive protein (mg/L)	0.8 (0.2–7.8)	1.9 (0.3–15.2)	<0.001
White blood cell (10^9^/L)	5.6(4.1–9.1)	6.6(3.9–14.6)	<0.001
Pancreatic Lipase (U/L)	33(5–201)	28(4–319)	<0.001
Amylase (U/L)	53 (7–122)	44(21–207)	<0.001
Free fatty acid (mmol/L)	0.45(0.11–0.90)	0.65(0.12–3.40)	<0.001
Hemoglobin (g/L)	139.0 ± 12.0	154.6 ± 13.3	<0.001
Platelet (10^9^/L)	212.5 ± 43.0	232.8 ± 51.0	<0.001
Red blood cell distribution width	12.1(11.5–15.9)	13.1(11.6–19.7)	<0.001

**Table 2:** Characteristics of the NAFLD Patients With and Without diabetes mellitus

Variable	diabetic NAFLD (n = 114)	non-diabetic NAFLD (n = 726)	*P* value
Age (yr)	45.3 ± 12.7	46.2 ± 12.3	0.301
Body mass index (kg/m^2^)	26.9 ± 2.3	26.2 ± 2.8	0.232
Systolic blood pressure (mmHg)	133.1 ± 17.1	131.6 ± 15.8	0.417
Diastolic blood pressure (mmHg)	81.1 ± 11.1	81.8 ± 37.2	0.709
Alanine aminotransferase (U/L)	32(9–138)	28 (6–184)	0.101
Aspertate aminotransferase (U/L)	27 (15–122)	24 (12–103)	0.095
Glutamyltransferase (U/L)	41(12–190)	33 (7–340)	0.041
Creatine Kinase (U/L)	92(39–330)	91(17–287)	0.215
Triglyceride (mmol/L)	1.98 (0.80–9.07)	1.61(0.34–20.30)	0.004
Total cholesterol (mmol/L)	5.12 ± 1.04	5.05 ± 0.97	0.295
High-density lipoprotein cholesterol (mmol/L)	0.98 ± 0.23	1.05 ± 0.27	0.091
Low-density lipoprotein cholesterol (mmol/L)	3.09 ± 0.80	2.97 ± 0.59	0.358
Fasting plasma glucose (mmol/L)	6.97(5.39–16.01)	5.02(3.39–6.01)	<0.001
Uric acid (umol/L)	384.7 ± 67.0	361.1 ± 81.4	0.014
Hemoglobin A1c (%)	7.2 (5.6–12.5)	5.5 (4.6–5.8)	<0.001
Homocysteine (umol/L)	16.3(5.3–150.3)	17.2(4.2–126.2)	0.241
Fe (umol/L)	24.1(9.4–52.4)	23.2(8.1–106.1)	0.346
Sialic acid (mg/dL)	78.6 ± 8.1	66.1 ± 6.7	<0.001
High-sensitivity C-reactive protein (mg/L)	2.0 (0.6–15.2)	1.9 (0.3–10.2)	0.079
White blood cell (10^9^/L)	6.9 (4.3–12.3)	6.5(3.9–14.6)	0.048
Pancreatic Lipase (U/L)	29 (4–186)	28(6–319)	0.194
Amylase (U/L)	36 (23–86)	45(21–207)	<0.001
Free fatty acid (mmol/L)	0.76(0.3–2.8)	0.62(0.12–3.40)	0.007
Hemoglobin (g/L)	156.6 ± 13.4	154.3 ± 13.3	0.602
Platelet (10^9^/L)	230.3 ± 56.5	233.2 ± 52.3	0.484
Red blood cell distribution width	13.7(11.6–18.2)	13.0(11.6–19.7)	0.785

**Table 3:** the odds values (95%CI) of Univariate regression analysis of dependent variables for predicting advanced fibrosis in NAFLD participants

Variable	Univariate	*P* value
Age	1.099 (1.082, 1.117)	<0.001
Body mass index	0.886 (0.815, 0.962)	0.004
Gender	0.560 (0.395, 0.793)	0.001
Alanine aminotransferase	1.005 (1.001, 1.010)	0.040
Aspertate aminotransferase	1.003 (0.999, 1.008)	0.125
Glutamyltransferase	1.005 (1.001, 1.008)	0.045
Creatine Kinase	1.001 (1.000, 1.003)	0.064
Triglyceride	1.110 (1.000, 1.206)	0.053
Total cholesterol	1.073 (0.909, 1.268)	0.403
Fasting plasma glucose	1.081 (0.952, 1.226)	0.229
Uric acid	1.001 (0.999, 1.003)	0.423
Homocysteine	0.998 (0.986, 1.010)	0.725
Fe	0.988 (0.970, 1.007)	0.212
High-sensitivity C-reactive protein	1.023 (1.002, 1.045)	0.043
presence of diabetes	0.718 (0.634, 0.785)	0.045
Free fatty acid	2.490(1.535, 4.039)	<0.001

**Table 4:** Independent predictors of advanced fibrosis in NAFLD participants according to free fatty acid in unadjusted and adjusted models

Model	Odds Ratio (95% CI)	P value
unadjusted	2.635 (1.482–4.684)	0.001
adjusted for age, gender, and Body mass index	3.460 (1.318–9.085)	0.011
adjusted for age, gender, Body mass index, Glutamyltransferase, and Alanine aminotransferase	2.934 (1.110–7.751)	0.029
adjusted for age, gender, Body mass index, Glutamyltransferase, Alanine aminotransferase, and High-sensitivity C-reactive protein	2.932 (1.109–7.753)	0.030
adjusted for age, gender, Body mass index, Glutamyltransferase, Alanine aminotransferase, High-sensitivity C-reactive protein and presence of diabetes	2.884(1.227–6.592)	0.031

Odds ratios were determined using logistic regression analyses.
